# A Novel Attitude Estimation Algorithm Based on the Non-Orthogonal Magnetic Sensors

**DOI:** 10.3390/s16050730

**Published:** 2016-05-19

**Authors:** Jianliang Zhu, Panlong Wu, Yuming Bo

**Affiliations:** Department of Automation, Nanjing University of Science and Technology, No. 200, Xiaolingwei Street, Xuanwu District, Nanjing 210094, China; zjl_njust@sina.com (J.Z.); byming@njust.edu.cn (Y.B.)

**Keywords:** extremum ratio method, integral ratio method, least square, magnetic sensor, projectile attitude

## Abstract

Because the existing extremum ratio method for projectile attitude measurement is vulnerable to random disturbance, a novel integral ratio method is proposed to calculate the projectile attitude. First, the non-orthogonal measurement theory of the magnetic sensors is analyzed. It is found that the projectile rotating velocity is constant in one spinning circle and the attitude error is actually the pitch error. Next, by investigating the model of the extremum ratio method, an integral ratio mathematical model is established to improve the anti-disturbance performance. Finally, by combining the preprocessed magnetic sensor data based on the least-square method and the rotating extremum features in one cycle, the analytical expression of the proposed integral ratio algorithm is derived with respect to the pitch angle. The simulation results show that the proposed integral ratio method gives more accurate attitude calculations than does the extremum ratio method, and that the attitude error variance can decrease by more than 90%. Compared to the extremum ratio method (which collects only a single data point in one rotation cycle), the proposed integral ratio method can utilize all of the data collected in the high spin environment, which is a clearly superior calculation approach, and can be applied to the actual projectile environment disturbance.

## 1. Introduction

The geomagnetic field is the earth’s natural resource, which can provide a natural coordinate system for aeronautics, astronautics and marine applications. The vehicle’s attitude information can be achieved by using magnetic detection technology to measure the components of the geomagnetic field. A projectile body rotates about its longitudinal axis at very high speed. Due to the limitation of the measuring range of the triaxial gyroscope, it is difficult to use the gyroscope in an actual high-speed, high-spin projectile environment. In contrast, a magnetic sensor has advantages such as a rapid response speed, small size, high resistance to overload, and being cumulatively error free [1,2,3]. A magnetic sensor is applicable to the high-speed, high-spin general projectile attitude measurement [4,5]. Projectile attitude information includes heading angle, pitch angle and rolling angle, and its characteristic is an approximately constant heading angle. Therefore, the projectile attitude information can be estimated using roll angle and pitch angle.

There are several methods for measuring the projectile attitude by unitizing magnetic sensors, including the two-axis non-orthogonal magnetic sensor, three-axis orthogonal magnetic sensor [6,7], and four-axis magnetic sensor. The non-orthogonal magnetic sensors [8] utilize two mounted non-orthogonal magnetic sensors to collect geomagnetic field data during one projectile spin cycle. Because the heading angle and pitch angle are nearly invariable when a projectile rotates one cycle, the roll angle of the projectile can be calculated directly. There are two non-orthogonal magnetic sensor methods for calculating the pitch angle of spinning projectiles: the zero crossing method [9,10] and the extremum ratio method [11]. Both methods utilize the relationship between the eigenvalue ratios of the sensor output and the pitch angle to calculate the projectile attitude. Both of these methods can obtain the estimated value of projectile attitude at a special point. The difference between these methods is as follows: the zero crossing method utilizes phase information from two sensors to calculate the projectile attitude [12], whereas the extremum ratio method utilizes extremum value information from two sensors to calculate the projectile attitude. However, because those two methods use the special point of the sampled magnetic data to calculate the attitude value as the projectile rotates one cycle, the two methods are vulnerable to random disturbance. As the error of sampling value becomes smaller, the error of the estimated projectile attitude becomes smaller, and vice versa [13]. The twin-channel tracking differentiator [14] method utilizes a differential filter to process data from the triaxial magnetic sensors, thereby reducing the random disturbance encountered by the projectile attitude.

To reduce the random disturbance that exists in the extremum ratio method, an integral ratio method is proposed in this paper. This method can reduce the random disturbance by establishing the integral model and can thus achieve greater attitude calculation accuracy than the extremum ratio method.

The remainder of this paper is organized as follows. In Section 2, the projectile attitude measurement principle of magnetic sensor is introduced. In Section 3, the mathematical model of integral ratio method is established. The analytical expression of integral ratio method is derived in Section 4. In Section 5, the simulation results demonstrate that the accuracy of the proposed algorithm is significantly higher than that of the extremum ratio method. Conclusions are drawn in Section 6.

## 2. Attitude Measurement Principle of the Magnetic Sensors

**Figure 1:**
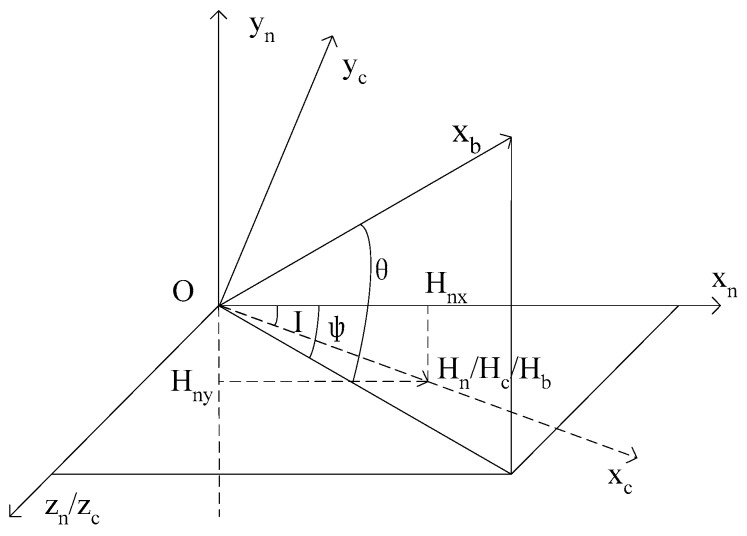
Coordinate transformation schematic diagram.

The geographical coordinate is $Ox_{n}y_{n}z_{n}$, the *x_n_* axis points to the magnetic north, the *y_n_* axis points to the sky, and the *Z_n_* axis points to the magnetic east. As shown in Figure 1, vector *H_n_* represents the magnitude and direction of the magnetic field in the geographical coordinate, *Ψ* is the heading angle, *θ* is the pitch angle, and *γ* is the roll angle.

The intersection angle between vector *H_n_* and its projection in horizontal plane *Px_n_z_n_* is geomagnetic inclination *I*. The carrier coordinate *Ox_c_y_c_z_c_* can be achieved by rotating geographical coordinate *Ox_n_y_n_z_n_* *I* degrees around the *z_n_* axis. The *x_c_* axis in *Ox_c_y_c_z_c_* coincides with the magnetic field vector *H_c_*, and *H_c_* represents the geomagnetic magnitude and direction of *Ox_c_y_c_z_c_*.

The instantaneous centroid of projectile is chosen as the origin of the projectile coordinate *Ox_b_y_b_z_b_*. The *Ox_b_* axis coincides with the lengthwise axis of projectile, pointing to the head, which is positive. *Oy_b_* is perpendicular to *Ox_b_*, and upward is positive. *Oz_b_* is perpendicular to *Ox_b_y_b_*, and the direction is determined by using the right hand rule. Vector *H_b_* represents the magnitude and direction of the magnetic field in the projectile coordinate *Ox_b_y_b_z_b_*.

The coordinates *H_bx_*, *H_by_*, *H_bz_* of vector *H_b_* in the projectile coordinate *Ox_b_y_b_z_b_* can be represented as the transformation from carrier coordinates to projectile coordinates $Ox_{c}y_{c}z_{c}\Rightarrow Ox_{n}y_{n}z_{n}\Rightarrow Ox_{b}y_{b}z_{b}$.

(1) $$\begin{array}{cc} H_{b} & =L(\gamma ,\psi ,\theta )L{\left(0,0,I\right)}^{T}H_{c}\\ & =L(\gamma ,\psi ,\theta -I)H_{c}\\ & =L(\gamma ,\psi ,\theta_{m})H_{c} \end{array}$$

where $\theta_{m}$ is the pitch angle, which includes magnetic dip, $\theta_{m}=\theta -I$; L(γ,ψ,θ) is the relationship that can be described by the direction cosine matrix.

(2) $$L(\gamma ,\psi ,\theta )=\left[\begin{array}{ccc} \cos\theta \cos\psi & \cos\psi \sin\theta & -\sin\psi \\ \sin\gamma \sin\psi \cos\theta -\cos\gamma \sin\theta & \sin\gamma \sin\theta \sin\psi +\cos\gamma \cos\theta & \sin\gamma \cos\psi \\ \cos\gamma \sin\psi \cos\theta +\sin\gamma \sin\theta & \cos\gamma \sin\theta \sin\psi -\sin\gamma \cos\theta & \cos\gamma \cos\psi \end{array}\right]$$

Vector *H_c_* in carrier coordinates *Ox_c_y_c_z_c_* is

(3) $$H_{c}={\left[\begin{array}{ccc} h & 0 & 0 \end{array}\right]}^{T}$$

where *h* is the magnitude of the vector *H_c_*.

The coordinates *H_bx_*, *H_by_*, *H_bz_* of vector *H_b_* in the projectile coordinates *Ox_b_y_b_z_b_* can be represented as [11]

(4) $$\left[\begin{array}{c} H_{bx}\\ H_{by}\\ H_{bz} \end{array}\right]=\;\left[\begin{array}{c} h\cos\theta_{m}\cos\psi \\ h(\sin\gamma \sin\psi \cos\theta_{m}-\cos\gamma \sin\theta_{m})\\ h(\cos\gamma \sin\psi \cos\theta_{m}+\sin\gamma \sin\theta_{m}) \end{array}\right]$$

**Figure 2:**
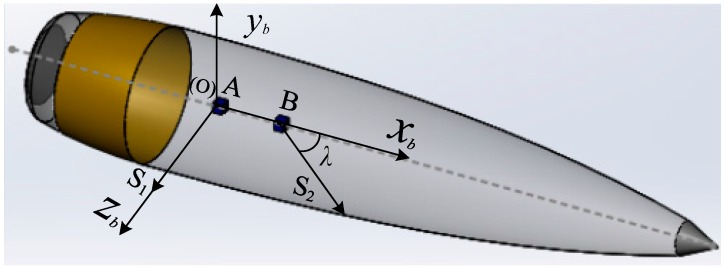
Schematic diagram of mounted magnetic sensors.

Two non-orthogonal single-axis magnetic sensors, S1 and S2, are assembled separately on A and B along with axis *Ox_b_* of the projectile. As shown in Figure 2, the axis *Ox_b_* coincides with the longitudinal axis of the projectile. Two sensitive axes are in the plane *Ox_b_y_b_*. The sensitive axis of S1 is assembled along with axis *Oz_b_*, and the sensitive axis of S2 is assembled in the angle of λ with axis *Ox_b_*. 

According to Equation (4), the measured data of magnetic sensor S1 form *H_bz_*, and the measured data of magnetic sensor S2 are the combination of *H_bx_* and *H_bz_* As a result, the measured data *H_s_*_1_ and *H_s_*_2_, of two magnetic sensors, can be expressed by $\theta_{m}$, γ and *h*:

(5) $$H_{S1}=h(\cos\gamma \sin\psi \cos\theta_{m}+\sin\gamma \sin\theta_{m})\;$$

(6) $$H_{S2}=h(\cos\theta_{m}\cos\psi \cos\lambda +\cos\gamma \sin\psi \cos\theta_{m}\sin\lambda +\sin\gamma \sin\theta_{m}\sin\lambda )\;\;$$

The projectile rotates at nearly constant speed during one spinning circle, and the heading angle *Ψ* and pitch angle *θ* are nearly invariable. The roll angles during one spinning cycle can be calculated by obtaining one roll angle at a particular time. Assuming that the particular time is the zero point time of *H_s_*_1_ and *H_s_*_2_, the unknown magnetic field intensity scalar *h* in Equations (5) and (6) can be eliminated when the measured data of *H_s_*_1_ or *H_s_*_2_ are zero. The effects of environmental disturbance can thus be reduced.

If the measured data *H_s_*_1_ = 0, then Equation (5) can be rewritten as

(7) $$\cos\gamma \sin\psi \cos\theta_{m}+\sin\gamma \sin\theta_{m}=0\;$$

Furthermore, we can obtain

(8) $$\gamma =\arctan2({\left(-1\right)}^{j+1}\sin\psi \cos\theta_{m},\;{\left(-1\right)}^{j}\;\sin\theta_{m})\;$$

where *j* = 1,2. The two components of Equation (8) cannot be zero simultaneously.

If the measured data *H_s_*_2_ = 0, then Equation (6) can be rewritten as

(9) $$\cos\theta_{m}\cos\psi \cos\lambda +\cos\gamma \sin\psi \cos\theta_{m}\sin\lambda +\sin\gamma \sin\theta_{m}\sin\lambda \;\;\;\;\;=0\;$$

If

(10) $$\left\{ \begin{array}{l} a=\cos\theta_{m}\cos\psi \cos\lambda \\ b=\sin\psi \cos\theta_{m}\sin\lambda \\ c=\sin\theta_{m}\sin\lambda \end{array}\right.$$

then

(11) a+bcosγ+csinγ    =0

When $\sin\theta_{m}\neq 0$, because sinλ≠0, then c≠0. Thus, Equation (11) can be described as

(12) $$\sin\gamma \;\;\;\;=\frac{-b\cos\gamma -a}{c}$$

Substituting Equation (12) into $\sin^{2}\gamma +\cos^{2}\gamma =1$ gives

(13) $$(b^{2}+c^{2})\cos^{2}\gamma +2ab\cos\gamma +a^{2}-c^{2}=0$$

When $\left|\sin\lambda \right|\geq \left|\cos\theta_{m}\cos\psi \right|$, ${\left(2ab\right)}^{2}-4(b^{2}+c^{2})(a^{2}-c^{2})\geq 0$, Equation (13) has real solution cosγ

(14) $$\cos\gamma \;=\frac{-a_{1}b_{1}\pm \sqrt{c_{1}{}^{2}(b_{1}{}^{2}+c_{1}{}^{2}-a_{1}{}^{2})}}{b_{1}{}^{2}+c_{1}{}^{2}}\;$$

where $a_{1}=\cos\theta_{m}\cos\psi \cos\lambda$, $b_{1}=\sin\psi \cos\theta_{m}\sin\lambda$, and $c_{1}=\sin\theta_{m}\sin\lambda$.

When $\left|\sin\lambda \right|>\left|\cos\theta_{m}\cos\psi \right|$, *H_s_*_2_ has two zero points.

When $\left|\sin\lambda \right|=\left|\cos\theta_{m}\cos\psi \right|$, *H_s_*_2_ has one zero point.

When $\left|\sin\lambda \right|<\left|\cos\theta_{m}\cos\psi \right|$, *H_s_*_2_ does not have a zero point.

From the above, we can see that the value of roll angle *γ* can be calculated from Equations (8) and (14) when the outputs of two magnetic sensors, S1 and S2, are equal to zero. There are four zero-crossing points that can be used individually to calculate the corresponding roll angle *γ*. Consequently, the roll angles at any time during one cycle can be calculated. Assuming that the heading angle *Ψ* does not change [15], when the included angle *λ* between magnetic sensor S2 and axis *Ox_b_* is given, the value of *γ* is related to the pitch angle $\theta_{m}$. As a result, to further ensure the value of the roll angle *γ*, it is important to utilize the magnetic data obtained as the projectile rotates one cycle to calculate $\theta_{m}$. Thus, assuming that the rotation speed remains constant, the calculation error of rolling angle is determined by the calculation error of the pitch angle as the projectile rotates one cycle. 

## 3. Mathematical Model

### 3.1. Mathematical Model of the Extremum Ratio Method

Assume that the heading angle and the pitch angle are invariable in one cycle. The extremum ratio method utilizes the characteristics that there exist one maximum value and one minimum value in every cycle. The pitch angle $\theta_{m}$ after the projectile rotates one cycle can be calculated. During one projectile rotation cycle, the roll angle changes between 0 and 2π. When the output curves *H_s_*_1_ and *H_s_*_2_ are at their extremum values, Equations (5) and (6) should meet the following conditions [6]:

(15) $$\frac{dH_{S1}}{d\gamma }=h(-\sin\gamma \sin\psi \cos\theta_{m}+\cos\gamma \sin\theta_{m})=0\;$$

(16) $$\frac{dH_{S2}}{d\gamma }=h(-\sin\gamma \sin\psi \cos\theta_{m}+\cos\gamma \sin\theta_{m})=0\;$$

The mathematic model of the integral ratio method can be described as

(17) $$g(\theta_{m})=\frac{H_{S2m}}{H_{S1m}}=\sin\lambda \pm \frac{\cos\theta_{m}\cos\psi \cos\lambda }{\sqrt{\sin^{2}\theta_{m}+\sin^{2}\psi \cos^{2}\theta_{m}}}\;$$

where *H_s_*_1*m*_ and *H_s_*_2*m*_ are the maximum or minimum values achieved by *H_s_*_1_ and *H_s_*_2_, respectively. $g(\theta_{m})$ represents the ratio of *H_s_*_1*m*_ and *H_s_*_2*m*_. If the heading angle *Ψ* and the included angle *λ* between S2 and axis $Ox_{b}$ are known, then the estimated pitch angle $\theta_{m}$ can be calculated by Equation (17).

### 3.2. Mathematical Model of the Integral Ratio Method

The mathematical model $g(\theta_{m})$ of the extremum ratio method utilizes the ratio between two particular points on magnetic sensor curves to calculate the pitch angle $\theta_{m}$. Its computational accuracy depends on the numerical precision of the sampling values *H_s_*_1_ and *H_s_*_2_ at the extreme point. Under practical conditions, due to various types of disturbance, some sampling values may have high error rates. In particular, if impulsive interference exists at any non-extreme point, a rather large sampling value will be generated. As a result, that value will be treated as the largest sampling value to calculate, thereby generating large errors.

A novel integral model is derived in this paper. During the rotation process, all of the samples of *H_s_*_1_ and *H_s_*_2_ are used to perform the integral calculation; subsequently, the ratio calculation is performed. The integral model is described as:

(18) $$f(\theta_{m})=\frac{\int_{0}^{2\pi }H_{S2}^{2}(\gamma )d\gamma }{\int_{0}^{2\pi }H_{S1}^{2}(\gamma )d\gamma }=\frac{f_{2}(\theta_{m})}{f_{1}(\theta_{m})}$$

The estimated value $\hat{f}(\theta_{m})$ of $f(\theta_{m})$ can be expressed as

(19) $$\hat{f}(\theta_{m})=\frac{\frac{2\pi }{N}\sum_{n=1}^{N}{\hat{H}}_{S2}^{2}(n)}{\frac{2\pi }{N}\sum_{n=1}^{N}{\hat{H}}_{S1}^{2}(n)}=\frac{{\hat{f}}_{2}(\theta_{m})}{{\hat{f}}_{1}(\theta_{m})}$$

where *N* is the total sampling number during one projectile rotation cycle and n is the sampling point. ${\hat{H}}_{S1}(n)=H_{S1}(n)+v_{1}(n)$, ${\hat{H}}_{S2}(n)=H_{S2}(n)+v_{2}(n)$, $H_{S1}(n)$ and $H_{S2}(n)$ are true value of magnetic sensors S1 and S2, respectively. ${\hat{H}}_{S1}(n)$ and ${\hat{H}}_{S2}(n)$ are sampled value of S1 and S2, respectively. $v_{1}(n)$ and $v_{2}(n)$ are both Gaussian white noise with a mean of zero and a variance of $\sigma^{2}$. In Equation (19), one value $\hat{f}(\theta_{m})$ can be calculated in every cycle using the quadratic sum and mean calculation with the sampling values from two magnetic sensors. 

The mathematical expectation of ${\hat{H}}_{S1}^{2}(n)$ can be described as

(20) $$E\left[{\hat{H}}_{S1}^{2}(n)\right]=H_{S1}^{2}(n)+E\left[v_{1}{\left(n\right)}^{2}\right]$$

where $v_{1}\sim N(0,\sigma^{2})$; according to the properties of normal distribution, we can get

(21) $${\left(\frac{v_{1}(n)}{\sigma }\right)}^{2}\sim \chi^{2}(1)$$

Therefore, $E\left[{\left(\frac{v_{1}(n)}{\sigma }\right)}^{2}\right]=1$, and

(22) $$E\left[{\left(v_{1}(n)\right)}^{2}\right]=\sigma^{2}$$

Substituting Equation (22) into Equation (20) gives

(23) $$E\left[{\hat{H}}_{S1}^{2}(n)\right]=H_{S1}^{2}(n)+\sigma^{2}$$

(24) $$D\left[{\hat{H}}_{S1}^{2}(n)\right]=E\left[{\hat{H}}_{S1}^{4}(n)\right]-E{\left[{\hat{H}}_{S1}^{2}(n)\right]}^{2}=4H_{S1}^{2}(n)\sigma^{2}$$

The mathematical expectation of ${\hat{f}}_{1}(\theta_{m})$ can be given by

(25) $$E\left[{\hat{f}}_{1}(\theta_{m})\right]=\frac{2\pi }{N}\sum_{n=1}^{N}\left(H_{S1}^{2}(n)+\sigma^{2}\right)=\frac{2\pi }{N}\sum_{n=1}^{N}H_{S1}^{2}(n)+2\pi \sigma^{2}$$

Similarly, the mathematical expectation of ${\hat{f}}_{2}(\theta_{m})$ can be given by

(26) $$E\left[{\hat{f}}_{2}(\theta_{m})\right]=\frac{2\pi }{N}\sum_{n=1}^{N}\left(H_{S2}^{2}(n)+\sigma^{2}\right)=\frac{2\pi }{N}\sum_{n=1}^{N}H_{S2}^{2}(n)+2\pi \sigma^{2}$$

Therefore, ${\hat{f}}_{1}(\theta_{m})$ and ${\hat{f}}_{2}(\theta_{m})$ are not unbiased estimators. Compared the mathematical expectation with unbiased estimation of ${\hat{f}}_{1}(\theta_{m})$ and ${\hat{f}}_{2}(\theta_{m})$, the magnitude of single sampling error variance is amplified 2π times. So the effect on parameter estimation is small and acceptable. ${\hat{f}}_{1}(\theta_{m})$ and ${\hat{f}}_{2}(\theta_{m})$ can be regarded as powers of two magnetic sensors’ sampled signal, and the power of the magnetic sensor signal is actually sampled as a characteristic value in the integral model to calculate the projectile attitude.

Thus, Equations (5) and (6) can be rewritten as

(27) $$H_{S1}=h(d_{2}\cos\gamma +e_{2}\sin\gamma )\;$$

(28) $$H_{S2}=h(a_{2}+b_{2}\cos\gamma +c_{2}\sin\gamma )\;$$

where $a_{2}=\cos\theta_{m}\cos\psi \cos\lambda$, $b_{2}=\sin\psi \cos\theta_{m}\sin\lambda$, $c_{2}=\sin\theta_{m}\sin\lambda$, $d_{2}=\sin\psi \cos\theta_{m}$, and $e_{2}=\sin\theta_{m}$.

The integral expression of $H_{S1}$ can be described as

(29) $$\begin{array}{cc} \int_{0}^{2\pi }H_{S1}^{2}(\gamma )d\gamma & =\int_{0}^{2\pi }{\left(h(d_{2}\cos\gamma +e_{2}\sin\gamma )\right)}^{2}d\gamma \;\\ & \;\;=h^{2}\int_{0}^{2\pi }(d_{2}{}^{2}\cos^{2}\gamma +e_{2}{}^{2}\sin^{2}\gamma +2d_{2}e_{2}\sin\gamma \cos\gamma )d\gamma \end{array}$$

Applying integral calculation on each items in Equation (29) separately gives

(30) $$\int_{0}^{2\pi }\cos^{2}\gamma d\gamma =\pi \;$$

(31) $$\int_{0}^{2\pi }\sin^{2}\gamma d\gamma =\pi \;$$

(32) $$\int_{0}^{2\pi }\sin\gamma \cos\gamma d\gamma =0\;$$

Substituting Equations (30)–(32) into Equation (29) gives

(33) $$\int_{0}^{2\pi }H_{S1}^{2}(\gamma )d\gamma =h_{2}{}^{2}(\pi d_{2}{}^{2}+\pi e_{2}{}^{2})\;$$

The integral expression of $H_{S2}$ can be described as

(34) $$\begin{array}{l} \int_{0}^{2\pi }H_{S2}^{2}(\gamma )d\gamma =\int_{0}^{2\pi }{\left(h(a_{2}+b_{2}\cos\gamma +c_{2}\sin\gamma )\right)}^{2}d\gamma \;\\ =h^{2}\int_{0}^{2\pi }(a_{2}{}^{2}+b_{2}{}^{2}\cos^{2}\gamma +c_{2}{}^{2}\sin^{2}\gamma +2b_{2}c_{2}\sin\gamma \cos\gamma +2a_{2}b_{2}\cos\gamma +2a_{2}c_{2}\sin\gamma )d\gamma \end{array}$$

Applying the integral calculation on each items in Equation (34) separately gives

(35) $$\int_{0}^{2\pi }\cos\gamma d\gamma =0\;$$

(36) $$\int_{0}^{2\pi }\sin\gamma d\gamma =0\;$$

Substituting Equations (30)–(32), (35) and (36) into Equation (34) gives

(37) $$\int_{0}^{2\pi }H_{S2}^{2}(\gamma )d\gamma =h^{2}(2\pi a_{2}{}^{2}+\pi b_{2}{}^{2}+\pi c_{2}{}^{2})\;$$

Using Equations (33) and (37), the integral model $f(\theta_{m})$ can be described as

(38) $$f(\theta_{m})=\frac{\int_{0}^{2\pi }H_{S2}^{2}(\gamma )d\gamma }{\int_{0}^{2\pi }H_{S1}^{2}(\gamma )d\gamma }\;=\frac{2\cos^{2}\theta_{m}\cos^{2}\psi \cos^{2}\lambda +\sin^{2}\psi \cos^{2}\theta_{m}\sin^{2}\lambda +\sin^{2}\theta_{m}\sin^{2}\lambda }{\sin^{2}\psi \cos^{2}\theta_{m}+\sin^{2}\theta_{m}}\;$$

Substituting $\sin^{2}\theta_{m}=1-\cos^{2}\theta_{m}$ into Equation (38) gives

(39) $$\cos^{2}\theta_{m}(2\cos^{2}\psi \cos^{2}\lambda +\sin^{2}\psi \sin^{2}\lambda -\sin^{2}\lambda -f(\theta_{m})\sin^{2}\psi +f(\theta_{m}))=f(\theta_{m})-\sin^{2}\lambda \;$$

We define $u=2\cos^{2}\psi \cos^{2}\lambda +\sin^{2}\psi \sin^{2}\lambda -\sin^{2}\lambda -f(\theta_{m})\sin^{2}\psi +f(\theta_{m})$ and $v=f(\theta_{m})-\sin^{2}\lambda$. Then, Equation (39) can be rewritten as

(40) $$uv\;\sin^{2}\theta_{m}=u(u-v)\cos^{2}\theta_{m}\;$$

The pitch angle $\theta_{m}$ is therefore given by

(41) $$\theta_{m}=\arctan2\left(\pm \sqrt{\left|u(u-v)\right|},\pm \sqrt{\left|uv\right|}\right)\;$$

In Equation (41), the two components cannot be zero simultaneously.

**Figure 3:**
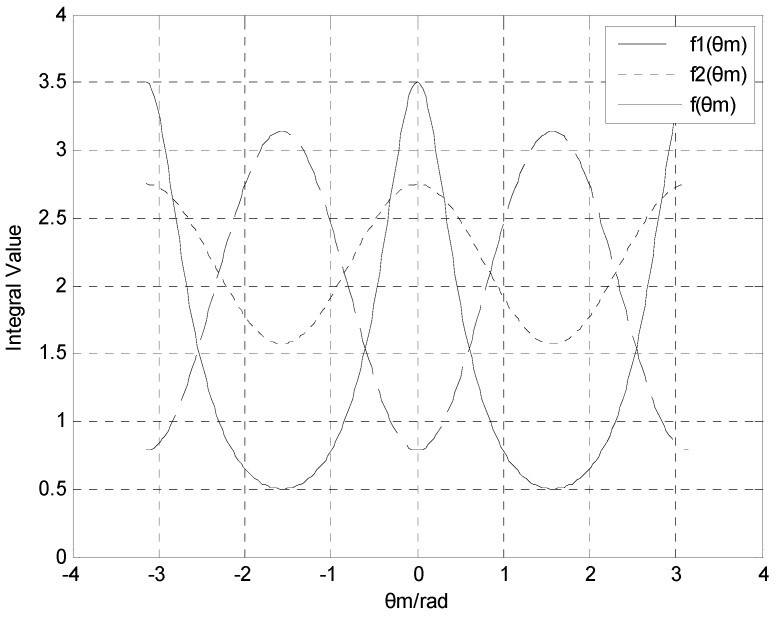
Comparison between the period of function ($f(\theta_{m})$) and the two integral outputs of feature values $f_{1}(\theta_{m})$ and $f_{2}(\theta_{m})$.

The period of pitch angle $\theta_{m}$ is 2π. The period of function $f(\theta_{m})$ with respect to pitch angle $\theta_{m}$ is π. As the projectile rotates one cycle, one $f(\theta_{m})$ value and the corresponding four $\theta_{m}$ values can be obtained. Assuming that h=1, Figure 3 gives the comparison between $f(\theta_{m})$ and the two integral outputs of $f(\theta_{m})$ and $f_{2}(\theta_{m})$. It can be seen that when the pitch angle of projectile takes any value, feature values ($f_{1}(\theta_{m})$ and$f_{2}(\theta_{m})$) can be got from integral models and the estimated value of $\theta_{m}$ can also be obtained throughout missile flight. However, one $f(\theta_{m})$ may correspond to two or four possible values of $\theta_{m}$, and the value of the pitch angle $\theta_{m}$ can be chosen based on the quadrant in which the pitch angle $\theta_{m}$ is located during the actual flight.

## 4. Novel Integral Ratio Method

**Figure 4:**
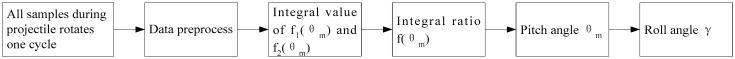
The flow chart of integral ratio method.

A novel integral ratio method is proposed in this paper, and the flow chart of this method is shown in Figure 4. Because this method utilizes all of the data collected during the projectile rotation process, it is convenient to preprocess those data. 

### 4.1. Data Preprocessing

Noise error exists in the measured data from the magnetic sensor. To reduce the noise disturbance, the filtering algorithm is usually adopted to preprocess the data smoothly. A maximum least squares filtering algorithm is used.

(1) When the samples number is less than the designated value *p*, the increasing memory filter is successively used to perform the filtering. *p* is chosen as 10 in this paper.
  (42) $$\hat{r}(k)=\frac{2(1-k)}{\left(k+1)(k+2\right)}\sum_{i=0}^{k}r(k-i)+\frac{6}{\left(k+1)(k+2\right)}\sum_{i=0}^{k}\left(k-i)r(k-i\right)\;$$
(2) When the sample number is greater than *p*, the fixed memory filter can be used. *p* is the filter order of moving average part.
  (43) $$\hat{r}(k)=\frac{2(1-p)}{\left(p+1)(p+2\right)}\sum_{i=k-p}^{k}r(k-i)+\frac{6}{\left(p+1)(p+2\right)}\sum_{i=k-p}^{k}\left(k-i)r(k-i\right)\;$$
  where r(k) is the sampling value at time k and $\hat{r}(k)$ is the least squares filtering value at time k.

### 4.2. The Principle of the Integral Ratio Method

Extreme ratio method only using a single extremum of sample data as characteristic value for processing, which is susceptible to random interference. But the improved integration ratio method utilizes the sum of all data squares of projectile spin as a characteristic value for processing, which can reduce the random noise on the estimated values. 

The implementation procedure of the proposed integral ratio method is given as follows:

Step 1: Preprocessing the data from the magnetic sensor using Equations (42) and (43).

The sampling values from the magnetic sensor are filtered by utilizing the maximum least squares filtering algorithm. After filtering the noise error, the value can be input into the integral model to calculate the model function $\hat{f}(\theta_{m})$.

Step 2: Calculating the integral value of $H_{S1}$ and $H_{S2}$.

1) Determine the integral expression $\frac{2\pi }{N}\sum_{n=1}^{N}{\hat{H}}_{S1}^{2}(n)$.
2) Determine the integral expression $\frac{2\pi }{N}\sum_{n=1}^{N}{\hat{H}}_{S2}^{2}(n)$.

Step 3: Calculating the integral ratio .$\hat{f}(\theta_{m})$.

One $\hat{f}(\theta_{m})$ value can be calculated according to Equation (19) by using the quadratic sum and the integral of the samples of two magnetic sensors.

Step 4: Calculating the pitch angle $\theta_{m}$.

During one projectile rotation cycle, assuming that both the included angle λ and the heading angle ψ are invariable, the pitch angle $\theta_{m}$ can be determined by Equation (41).

Step 5: Calculating the roll angle γ using Equations (8) and (14).

## 5. Simulation and Results

### 5.1. Comparison of the Algorithms

**Figure 5:**
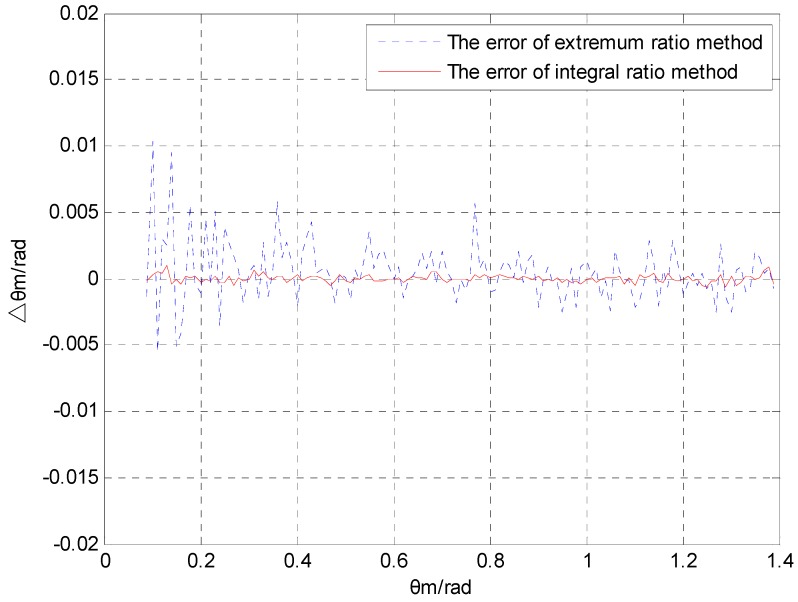
Comparison of the pitch angle error with $\sigma^{2}=0.001$.

**Figure 6:**
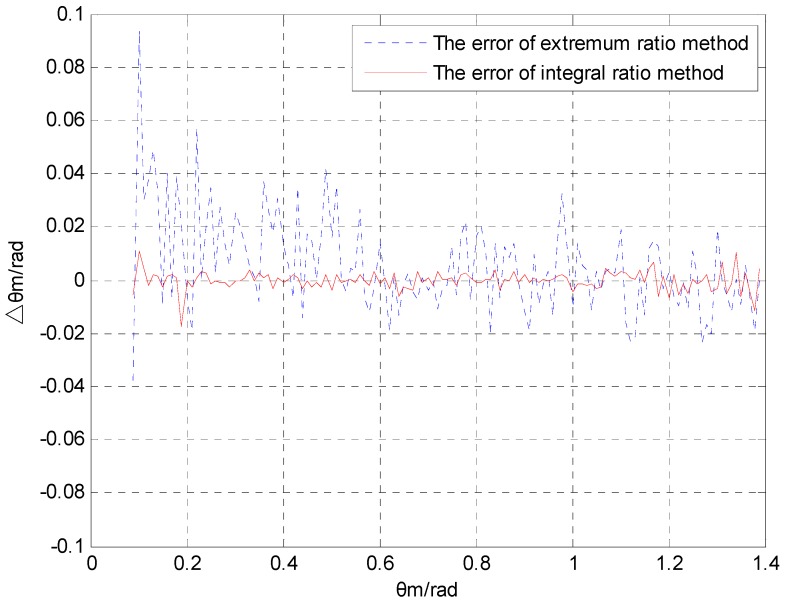
Comparison of the pitch angle error with $\sigma^{2}=0.001\;$.

In this section, the noise restraining abilities of the extremum ratio method and the proposed integral ratio method are compared to each other. Assuming that the projectile’s heading angle is ψ=π/6, the included angle between magnetic sensor S2 and axis $Ox_{b}$ is λ=π/4, and the data range of pitch angle is $\theta_{m}\in (0,\pi /2)$. The noise of the magnetic sensor is zero mean white noise. Figure 5 and Figure 6 show a comparison of the pitch angle error $\Delta \theta_{m}$ for different noise intensities ($\sigma^{2}=0.001$ and $\sigma^{2}=0.01$, respectively) after applying the extremum ratio and the proposed integral ratio methods. 

Figure 5 and Figure 6 show that both algorithms have rather large errors around $\theta_{m}=0$. The reason for the error is that the numerical difference of the magnetic component is too small near the zero point and the error of extremum is too large. The calculation error of the pitch angle also depends on the noise intensity. The error during the variance 0.01 is one order of magnitude greater than the error observed with a variance of 0.001. In the range of the pitch angle, the calculation error of the integral ratio method is much smaller than that of the extremum ratio method. 

Table 1 shows the mean and variance of the pitch angle errors, which were calculated by applying the extremum ratio method and the integral ratio method, as well as the error comparison between these methods under different noise intensities. When the noise variance is 0.001, the mean and variance of the errors by applying integral ratio method can be reduced by 84.6% and 89.2%, respectively, compared with that of the errors obtained by applying the extremum ratio method. When the variance of noise is 0.01, the mean and variance of the errors by applying integral ratio method can be reduced by 90.2% and 96.1%, respectively, compared with that of the errors obtained by applying the extremum ratio method.

Table 1 indicates that the mean and variance of the errors obtained by applying the integral ratio method are significantly smaller than those of the errors obtained by applying the extremum ratio method. In addition, as the random errors from the magnetic sensor become larger, the integral ratio method becomes clearly superior in terms of calculation accuracy. Thus, the integral ratio method is clearly superior when the magnetic sensor operates under an environment with a high level of disturbance. 

The extremum ratio method utilizes the extremum to calculate the pitch angle as the projectile rotates one cycle. If the extremum encounters any disturbance, the calculation will produce errors. However, the integral ratio method uses the result of the square of the magnetic sensor output to perform the integral calculation. Because the integral calculation performs the function of a filter when the data encounters any white noise, the integral ratio method has strong noise suppression ability. 

From the above-mentioned information, we can see that the proposed integral ratio method is clearly superior in both the mean value and the variance of the calculation error compared to the extremum ratio method. When the variance of random noise is 0.01, the error of the integral ratio method is only 10% of the error of the extremum ratio method.

### 5.2. Ballistic Simulation

The dynamic model of the projectile with magnetic sensor assembled is described as

(44) $$\left\{ \begin{array}{l} \frac{dx}{dt}=v_{x}\\ \frac{dy}{dt}=v_{y}\\ \frac{dz}{dt}=v_{z}\\ \frac{dv_{x}}{dt}=-cH_{\tau }(y)G(v_{\tau })v_{x}\\ \frac{dv_{y}}{dt}=-cH_{\tau }(y)G(v_{\tau })v_{y}-g\\ \frac{dv_{z}}{dt}=-cH_{\tau }(y)G(v_{\tau })v_{z} \end{array}\right.\;$$

where *x*, *y* and *z* are the position components and $v_{x}$, $v_{y}$ and $v_{z}$ are the velocity components.

The ballistic coefficient $c=(iD^{2}/m)\times 10^{3}$, *D* is the diameter of projectile, *i* is the elastic coefficient, and m is the mass of projectile. 

$H_{\tau }(y)=H_{\rho }(y)\sqrt{\tau /\tau_{on}}$, $\tau_{on}$ is the standard virtual temperature. $\tau =\tau_{on}-0.0065h_{f}$ is the virtual temperature, $H_{\rho }(y)=\rho /\rho_{on}$ is the function of air density, $\rho_{on}=1.225\;$kg/m^3^, $\rho =\rho_{on}\exp\left(-gh_{f}/\left(287.15\tau \right)\right)$, and $h_{f}$ is the flight height.

$G(v_{\tau })$ is the resistance function, and $v_{\tau }=\sqrt{\left(v_{x}^{2}+v_{y}^{2}+v_{z}^{2}\right)\left(\tau_{on}/\tau \right)}$.

$g=g_{0}\left(1-2h_{f}/\left(R_{0}+h_{f}\right)\right)$ is the gravitational acceleration, $g_{0}=9.780\;$m/s^2^, and $R_{0}=6378160\;$m.

The equations of the projectile attitude is denoted as

(45) $$\left\{ \begin{array}{l} \psi =\arctan\frac{v_{z}}{v_{x}}\\ \theta =\arctan\frac{v_{y}}{v_{x}}\\ \gamma =-\frac{\omega_{g}J}{0.4LD^{3}}\exp\left(-0.4\frac{LD^{3}}{J}t\right) \end{array}\right.\;$$

The initial conditions of the flight trajectory are set as follows: *D* = 152 mm, *L* = 1300 mm, *m* = 52.8 kg, $\omega_{g}=40\pi \;$rad/s, and *J* = 7.5 kg·m^2^. The muzzle velocity is 550m/s, the initial heading angle is ψ=π/6, the pitch angle is $\theta_{m}=\pi /4$, and the rolling angle is γ=0. The included angle between the pitch angle of magnetic sensor S2 and axis $Ox_{b}$ is λ=π/4. The noise of the magnetic sensor is white noise, with a mean of 0 and a variance of 0.01 or 0.001. Both the magnetic sensor sampling period and the updating period of the projectile attitude are 1 ms.

**Figure 7:**
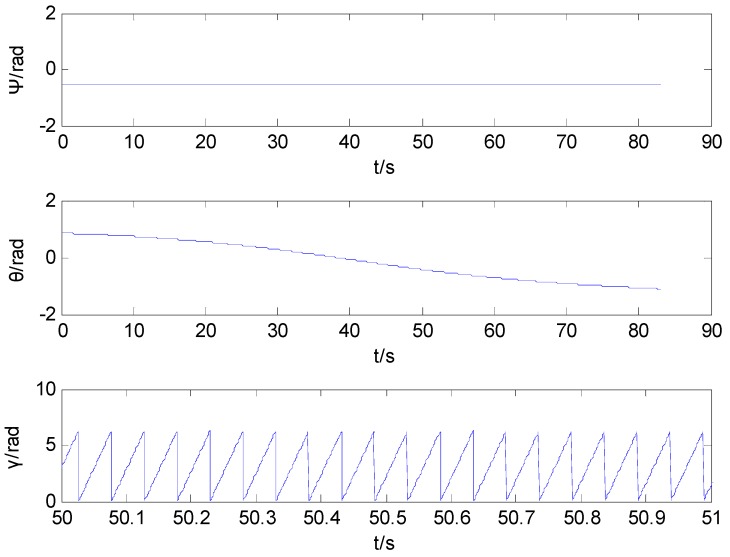
The curves of the attitude angle during projectile flight.

Figure 7 shows the curves of the heading angle, pitch angle and rolling angle. The simulation curve of the rolling angle runs only 1 s, from 50 s to 51 s.

**Figure 8:**
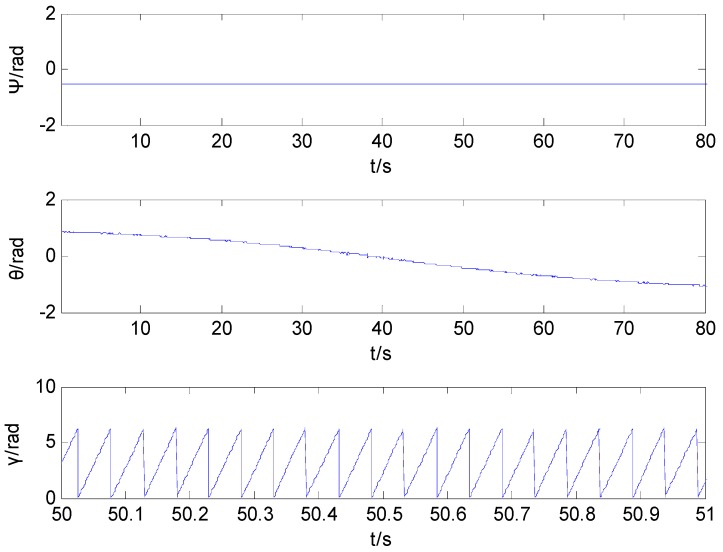
The attitude angle calculated by the integral ratio method.

**Figure 9:**
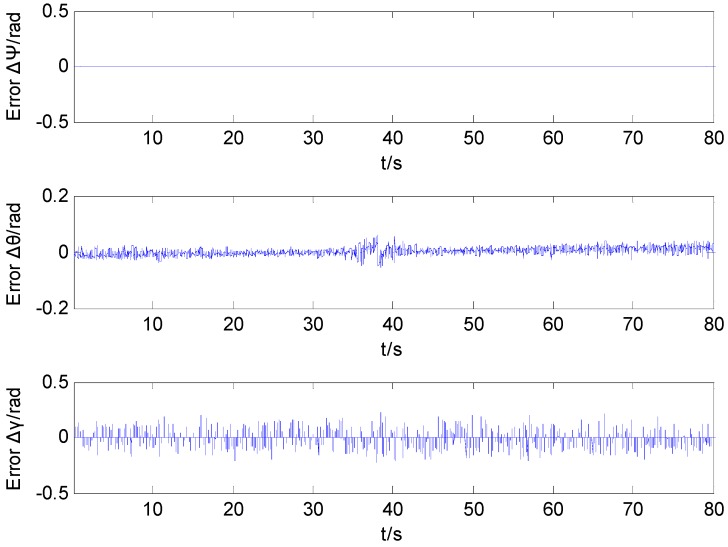
Attitude angle calculating error of the integral ratio method.

Figure 8 shows the curve of the projectile attitude obtained by utilizing the proposed integral ratio method. The simulation curve of the rolling angle runs only 1 s. The result shows that the proposed integral ratio method can produce an estimated value of the projectile attitude during the whole trajectory. Figure 9 shows the error curve of the projectile attitude by utilizing the integral ratio method.

**Figure 10:**
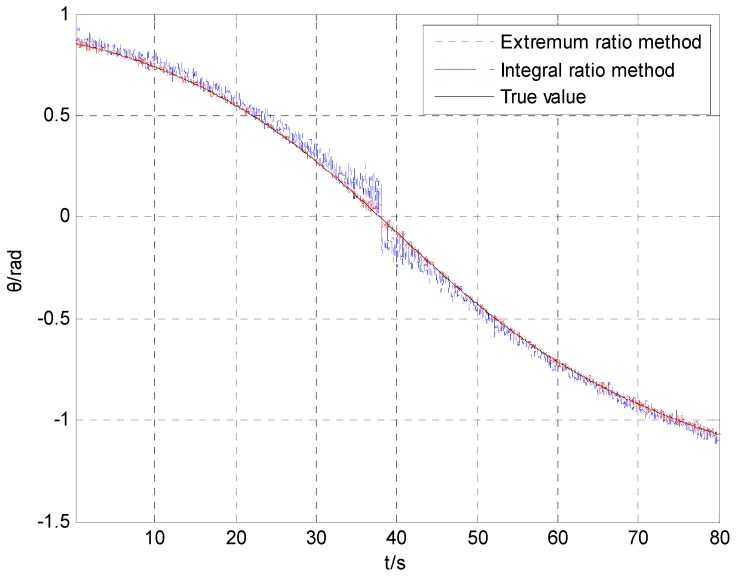
The pitch angle calculated by the extremum ratio method and the integral ratio method.

Figure 10 indicates that the calculation error of the pitch angle obtained by utilizing the integral ratio method is much smaller than that obtained by utilizing the extremum ratio method.

Table 2 lists the variance of the calculation error of the pitch angle and the rolling angle obtained by utilizing two different methods. The data show that the variance of calculation error of pitch angle obtained by utilizing the integral ratio method is one order of magnitude smaller than that obtained by utilizing the extremum ratio method. The variance of the calculation error of the rolling angle obtained by utilizing the integral ratio method is only half that obtained by utilizing the extremum ratio method.

## 6. Conclusions

To solve the computational problem of a projectile’s pitch angle, the extremum ratio method can be used to select the extreme point to perform the calculation. According to the problem relating to the fact that the extremum ratio method will result in a large error under a noise disturbance, a novel integral ratio method was proposed, and the corresponding mathematical model was derived. In this paper, the computational expression of pitch angle was derived by utilizing the integral ratio method. The simulation results provided comparison diagrams between the extreme ratio method and the integral ratio method under different noise intensities. Compared to the extremum ratio method, the integral ratio method has several advantages. When the variance of noise becomes larger, the integral ratio method is clearly superior in terms of the calculation error. In addition, the extremum ratio method only selects one data point in every spin cycle, whereas the integral ratio method selects multi-group data in every spin cycle; as a result, multiple data processing algorithms can be performed to get more accurate attitude estimation. Furthermore, the integral model allows for digital processing. An existing digital device can be utilized to perform high-speed, high-precision AD (analog digital conversion) sampling. 

## Figures and Tables

**Table 1:** The comparison of the pitch angle error.

Method	Error Mean		Error Variance	
	(unit: 0.001 rad)		(unit: 0.001)	
	σ^2^ = 0.001	σ^2^ = 0.01	σ^2^ = 0.001	σ^2^ = 0.01
Extremum ratio method	0.45389	11.2	0.017722	0.87621
Integral ratio method	0.070004	1.1	0.0019182	0.034168
Error reduction	84.6%	90.2%	89.2%	96.1%

**Table 2:** The error variance comparison between the extremum ratio method and the proposed integral ratio method.

Method	Pitch Angle	Roll Angle
	(unit: 0.001)	(unit: 0.001)
Extremum ratio method	1.4526	0.20321
Integral ratio method	0.18652	0.093807
Error reduction	87.2%	53.8%
